# Identification of candidate genes involved in root gall formation during early infection of *Plasmodiophora brassicae* in *B.napus*

**DOI:** 10.3389/fpls.2026.1847458

**Published:** 2026-06-10

**Authors:** Yujun Xue, Wenjie Guan, Fang Qian, Guangqin Cai, Chunyu Zhang, Xiaoming Wu

**Affiliations:** 1 The Key Laboratory of Biology and Genetic Improvement of Oil Crops, the Ministry of Agriculture and Rural Affairs of the People's Republic of China (PRC), Oil Crops Research Institute of the Chinese Academy of Agricultural Sciences, Wuhan, China; 2 National Key Laboratory of Crop Genetic Improvement and College of Plant Science and Technology, Huazhong Agricultural University, Wuhan, China

**Keywords:** *Brassica napus*, candidate genes, clubroot disease, GWAS, transcriptome, visual phenotyping

## Abstract

Clubroot disease, caused by *Plasmodiophora brassicae*, is one of the major constraints in rapeseed production. Breeding disease-resistant cultivars is the best way to control this devastating disease. However, breeding reliable resistant germplasm and genes is limited. Inactivation of susceptible genes has been shown to be a new and effective strategy for developing resistant crops. Therefore, we aimed to screen key candidate susceptible genes in this study. Firstly, we established a stable, high-throughput visualization method for identifying gall formation at the early stage of *P.brassicae* infection. At 14 days post-inoculation (dpi), the earliest time point with a clear record of scorable root swelling, remarkable variations in the speed of gall formation were observed among 85 genotypes. Secondly, genome-wide association studies (GWAS) were performed to identify genes involved in gall development. Three and two consecutive significant peaks were detected at 14 and 21 dpi, respectively. Thirdly, comparative transcriptomic analysis was conducted between 2AF195 and 2AF058 at 7 and 14 dpi; these two materials exhibit contrasting speeds of gall development. Gene clustering analysis revealed two opposite expression patterns at 14 dpi. One pattern comprised 1,383 genes downregulated in 2AF195 but upregulated in 2AF058, which were significantly enriched in 10 KEGG pathways, including Environmental Information Processing and Plant-pathogen interaction, and involved core repressors JAZ8/10 in the jasmonic acid (JA) signaling pathway, as well as nucleotide-binding site (NBS) protein-encoding genes. The opposite pattern consisted of 79 genes upregulated in 2AF195 but downregulated in 2AF058, which were enriched in an additional 10 KEGG pathways, predominantly related to Carbohydrate Metabolism and the Ubiquitin System. These genes were functionally annotated mainly as pectin methylesterases, xyloglucan endotransglucosylase/hydrolases (XTHs), and lignin biosynthesis-related enzymes. These findings demonstrated that distinct regulatory networks exist in different susceptible rapeseed genotypes. Finally, through the combined analysis of haplotype and transcriptome data, we co-localized and identified the candidate gene *BnaC08g46100D*, a nodulin-related gene belonging to the MtN21 transporter family. These results provide a theoretical basis for developing novel disease-resistant materials by editing the key susceptibility genes involved in root gall formation. The candidate genes identified in this study are the most promising targets for this purpose.

## 1 Introduction

Clubroot is a devastating disease of cruciferous crops, including rapeseed (*Brassica napus* L.), cabbage (*Brassica rapa*), cauliflower (*Brassica oleracea*), radish *(Raphanus sativus*), etc., which is caused by the soil-borne protist *Plasmodiophora brassicae*. This disease can lead to yield losses of 20%-30% in susceptible fields and may cause complete crop loss in severe outbreaks (Chai et al., 2014; Gill and Dhillon, 2022). Clubroot disease can be managed through various methods, but the most economical and efficient strategy remains the breeding of resistant cultivars by transferring clubroot-resistant genes into cultivars.

Numerous studies have cloned and characterized several resistance genes, including *CRa* (also known as CRA3.7.1), *Crr1a*, and *CRA8.2.4*, all of which encode typical TNL (Toll/interleukin-1 receptor, nucleotide-binding site, leucine-rich repeat) proteins (Ueno et al., 2012; Hatakeyama et al., 2013; Yang et al., 2022). Another functionally characterized gene, *WTS* (WeiTsing), encodes an endoplasmic reticulum-localized protein whose expression triggers a calcium-mediated immune response and cell death, thereby conferring disease resistance to plants (Wang et al., 2023). To date, approximately 39 major clubroot resistance loci have been reported, most of which are dominant genes with race-specificity (Lai et al., 2025). Due to the high heterogeneity of *P. brassicae* populations in agricultural fields, multiple physiological races can easily emerge (Williams, 1966; Buczacki et al., 1975; Hollman et al., 2020; Zamani-Noor et al., 2022; Zeng et al., 2024). For instance, in China, 26 distinct physiological races have been identified from 33 field isolates (Zeng et al., 2024). In practice, single-gene-resistant cultivars that target only specific physiological races often lose their resistance after 4–5 years of cultivation (Strelkov et al., 2016; Strelkov, 2018; Hollman et al., 2020). Relying on a single dominant resistance gene or pyramiding minor-effect quantitative trait loci for resistance typically fails to achieve durable resistance (Agyeman et al., 2018), resulting in a lack of stable, broad-spectrum resistance in existing varieties. Therefore, it is crucial to exploit an alternative way to develop novel resistant crops. With the fast advancement of gene editing technology, such as clustered regularly interspaced short palindromic repeats (CRISPR)/CRISPR-associated protein 9 (Cas9) (CRISPR/Cas9), inactivation of susceptible genes has been proven to be a new and effective strategy to develop resistant crops (Hu et al., 2024; Wu Q. et al., 2025). *GSL5* (β-1,3-glucan synthase-like protein 5) is closely associated with clubroot susceptibility. Loss-of-function mutations in GSL5 can confer broad-spectrum, high-efficiency resistance against multiple *P.brassicae* pathotypes in crops such as *B.napus*, *Brassica rapa*, and *Brassica oleracea* (Wu Y. et al., 2025). In tomato (*Solanum lycopersicum*) and *Arabidopsis* (*Arabidopsis thaliana*), loss-of-function mutations in the TOPLESS family-specific member TPL1 significantly reduce plant susceptibility to *Fusarium oxysporum* (Aalders et al., 2024). In banana (Musa spp.), disease resistance research and gene expression analysis, CRISPR/Cas9-mediated editing of the *MusaENODL3* gene conferred resistance to bacterial wilt (BXW) in banana plants (Ntui et al., 2024). Therefore, elucidating the infection process of *P. brassicae* in susceptible hosts is crucial. Research by Prof. Liu indicated that days 1–7 post-inoculation with *P. brassicae* constitute the primary infection stage in root epidermal tissues, during which secondary zoospores are formed. By day 8 post-inoculation, numerous uninucleate secondary plasmodia appear in cortical cells (marking the establishment of secondary infection), followed by the secondary infection stage in root cortical tissues from days 8 to 24 (Ingram and Tommerup, 1972; Liu, 2020).

Therefore, silencing key susceptibility genes has become a new and effective approach for developing disease-resistant germplasm, which is particularly important and useful for crops with scarce disease-resistant gene resources.

*B.napus* is the world’s largest oil crop and an important source of oil and protein. As a new allopolyploid crop that has only formed in about 10,000 years (Chalhoub et al., 2014), the resources of clubroot-resistant genes are extremely scarce in this species. All the clubroot-resistant genes in currently bred resistant varieties are derived from *B.rapa* through interspecific hybridization (Diederichsen et al., 2009; Hasan and Rahman, 2016). Therefore, silencing susceptibility genes in *B.napus* is expected to provide more and better disease-resistant germplasm for breeding resistant rapeseed cultivars.

The key to adopting this strategy is to identify the critical stages of clubroot development and the key susceptibility genes involved. However, clubroot develops on the roots, which grow underground, and existing disease resistance phenotyping methods cannot observe the disease development stages in a real time manner, nor the phenotypic differences among varieties during these critical stages, because conventional clubroot resistance evaluation is typically conducted six weeks after sowing: plants are uprooted from the soil, their root systems are thoroughly washed, and root phenotypes are observed (Tomita et al., 2013; Strelkov, 2018; Yang et al., 2020). At this stage, the clubroot galls have already grown to a considerable size, yet the differences between different genotypes are difficult to distinguish, and the exact time when the galls appear cannot be determined.

To address the above issues, this study developed a transparent visual phenotyping method for real-time identification of root gall formation and development. Using this method, we identified the critical time points of clubroot gall emergence in 85 accessions of rapeseed mini core germplasm and the differences in gall emergence time among these varieties. Based on observed phenotypic variation, a genome-wide association study (GWAS) and transcriptome analysis were employed to identify candidate genes and gene regulatory networks associated with clubroot gall formation. The findings of this study provide a theoretical basis and candidate target susceptibility genes for the development of clubroot-resistant germplasm by gene editing. It is expected to address the challenge of the scarcity of disease-resistant genes in *B. napus*.

## 2 Materials and methods

### 2.1 Plant material and pathogen isolate

In this study, 85 core rapeseed germplasm resources, including a slow gall-developing material 2AF195 and a fast gall-developing material2AF058, were obtained from the National Medium-term Gene Bank of the Oil Crops Research Institute, Chinese Academy of Agricultural Sciences (OCRI-CAAS) (**Supplementary Table 1**). These resources are part of the 418 *B.napus* germplasm reported in a previous publication (Hu et al., 2022). The disease-resistant material 317 (resistant control, CK1) and the susceptible material ZS11 (susceptible control, CK2), previously identified, were used as experimental control materials.

The *P.brassicae* used was Xinmin (*Pb*XM), China, pathotype 4 based on Sinitic Clubroot Differentiation (SCD) classification. The preparation methods for the resting spore suspension and the infested soil followed those described in a previous study (Li et al., 2016). The suspension was diluted with sterile water to a concentration of 3×10^8^ spores/mL and stored at 4 °C.

### 2.2 Plant cultivating and clubroot phenotyping

Eighty-five germplasm resources, along with the cultivars 317 and ZS11, were used. Multiple plump seeds of each were germinated in square Petri dishes lined with two layers of absorbent paper. Germination and initial growth were conducted for 7 days in an artificial climate chamber set at 22 °C/19 °C (16 h light/8 h dark). A uniformly mixed inoculum-soil substrate was then prepared and used to fill cultivation boxes. Seedlings were transplanted into this substrate, which contained 10^6^ resting spores per gram of soil. Cultivation conditions remained identical to those in the climate chamber. Five varieties were planted per transparent box, with three plants per variety. Control materials were planted with one plant each. The experiment was conducted with three biological replicates.

The development of clubroot symptoms on rapeseed roots was monitored continuously through the transparent boxes. The number of plants exhibiting clearly discernible root swelling was recorded. The Ratio of Visible Clubroot (RVC) was calculated as the proportion of plants showing clear swelling among the total of 9 plants (3 plants × 3 replicates). Materials were photographed at 7, 14, 21, 28, and 35 days post-inoculation (dpi).

Two materials-2AF195 (asymptomatic at 14 dpi) and 2AF058 (symptomatic at 14 dpi)-were separately planted in the control group (CK, watered) and treatment group (T, inoculated with the *Pb*XM), with cultivation methods and conditions as described above. Root tissues were collected at 7 days and 14 days after planting/transplantation. Samples were immediately frozen in liquid nitrogen and stored at -80 °C for subsequent use.

### 2.3 Population structure and linkage disequilibrium analysis

Genotypic data of 85 *B. napus* germplasm accessions were retrieved from a previously reported set of 418 accessions (Hu et al., 2022). VCFtools (v.0.1.16) was used to filter 5,287,597 high-quality single-nucleotide polymorphism (SNP) loci based on the criteria: minor allele frequency (MAF)≥0.05, missing rate ≤ 0.2, and sequencing depth ≥ 6 (Danecek. Subsequently, VCF2Dis (v.1.47), the FastME 2.0 webserver (http://www.atgc-montpellier.fr/fastme/), and the iTOL webserver (https://itol.embl.de/) were sequentially employed to calculate genetic distances, generate distance matrices, and construct a phylogenetic tree (Lefort. We inferred population structure (Q) from the genotypic data using ADMIXTURE (v1.3.0), setting the number of subpopulations (K) to the minimum cross-validation error (CV) (Alexander et al., 2009). Furthermore, GEMMA (v.0.94.1) was applied to generate the kinship matrix (K) from phenotypic and genotypic data (Zhou and Stephens, 2012). Linkage disequilibrium (LD) decay analysis was performed for all 85 accessions, as well as the semi-winter, winter, and spring subpopulations, using PopLDdecay (v.3.41) software (Zhang et al., 2019). The physical distance at which the squared correlation coefficient of allele frequencies (r^2^) decreased to 0.1 was defined as the LD decay distance. The results showed that the LD decay distance of the 85 accessions was approximately 113 kb.

### 2.4 Genome-wide association study

A genome-wide association study (GWAS) was conducted in GEMMA (v0.94.1) to associate resequencing-based genotypes of 85 accessions with their RVC data at 14 and 21 dpi, applying a mixed linear model (MLM; P + K model). QQ plots and Manhattan plots of the GWAS results were generated using the qqman package in R (Turner, 2014). A total of 562,643 independent and effective SNPs were screened using PLINK with the parameter --indep -pairwise 100 5 0.2. Based on the Bonferroni correction, the genome-wide significance threshold was calculated as P = 1/n, where n represents the number of independent effective SNPs. The significance threshold was set at 1.7773×10^−6^ (corresponding to -log_10_(P) = 5.75) to identify reliable marker-trait association loci. To validate the authenticity of the identified SNPs, significance analysis of phenotypic differences associated with alleles at the associated loci was conducted using SPSS (v.22.0; IBM Corp., Armonk, NY, USA), and SNPs showing no significant differences were removed (Turner, 2014). The QTL interval was defined as a 150 kb genomic region extending to both sides of each significantly associated SNP, based on the LD decay distance of approximately 113 kb with appropriate expansion. LD blocks containing significant associated SNPs were identified using PLINK (v.1.90) to prioritize candidate genes potentially affected by the target SNPs (Purcell et al., 2007).

### 2.5 Transcriptomic data analysis

RNA sequencing libraries were prepared from 10 mixed-pool: 2AF195 and 2AF058 materials treated with *Pb*XM and their corresponding controls at 7 and 14 dpi. A total of 5 μg of RNA per sample was used following the standard Illumina protocol. Library construction and sequencing were performed by Berry Genomics Co., Ltd. in Beijing, China, using the Illumina PE150 platform. The RNA-seq samples were designated as follows: CK-7D-2AF195, T-7D-2AF195, CK-7D-2AF058, T-7D-2AF 058, CK-14D-2AF195, T-14D-2AF195, CK-14D-2AF058, and T-14D-2AF058. Here, “7D” and “14D” represent samples collected at 7 and 14 dpi, respectively, while “CK” and “T” denote the application of water (control) and *P. brassicae* (treatment), respectively.

Raw sequencing data were subjected to quality control. Subsequently, the cleaned reads were aligned to the reference genome using HISAT2 (v2.2.1) with default parameters (Gill and Dhillon, 2022). Differential expression analysis was performed using the DESeq2 software (v1.34.0) via the edgeR package (v3.40.0) in R. Differentially expressed genes (DEGs) were identified with a threshold of false discovery rate (FDR)<0.05 and |log2 fold change (FC)|≥1 (Liu S. et al., 2021). Volcano plots were generated using the ggplot2 package (v3.4.4) in R. The identified DEGs were then subjected to Gene Ontology (GO) functional enrichment analysis (Ashburner et al., 2000) and Kyoto Encyclopedia of Genes and Genomes (KEGG) pathway enrichment analysis (Kanehisa et al., 2021).

### 2.6 Haplotype analysis and candidate genes screening

Utilize the genomic information and annotation files of the *Darmor* reference genome in the BnIR database (https://yanglab.hzau.edu.cn/BnIR/) developed by Huazhong Agricultural University to retrieve the gene information within the associated QTL regions, and conduct sequence alignment on the Arabidopsis genome to obtain homologous genes and their annotation information, and then screen out the candidate genes related to clubroot disease within the refined QTL regions (Clark et al., 2007; Chalhoub et al., 2014; Liu D. et al., 2021). Use the LD BlockShow software to perform haplotype analysis of the QTL segments associated with candidate genes for clubroot disease and draw an LD heat map (Dong et al., 2021). Utilize the SPSS 22.0 software to conduct a significant difference analysis (with significance level α < 0.05) on the phenotypes corresponding to the haplotypes of the QTL segments where the candidate genes are located, so as to verify the candidate genes and obtain the haplotypes of the target genes.

## 3 Results

### 3.1 Development of real-time phenotyping method for observation of clubroot

**Figure 1:**
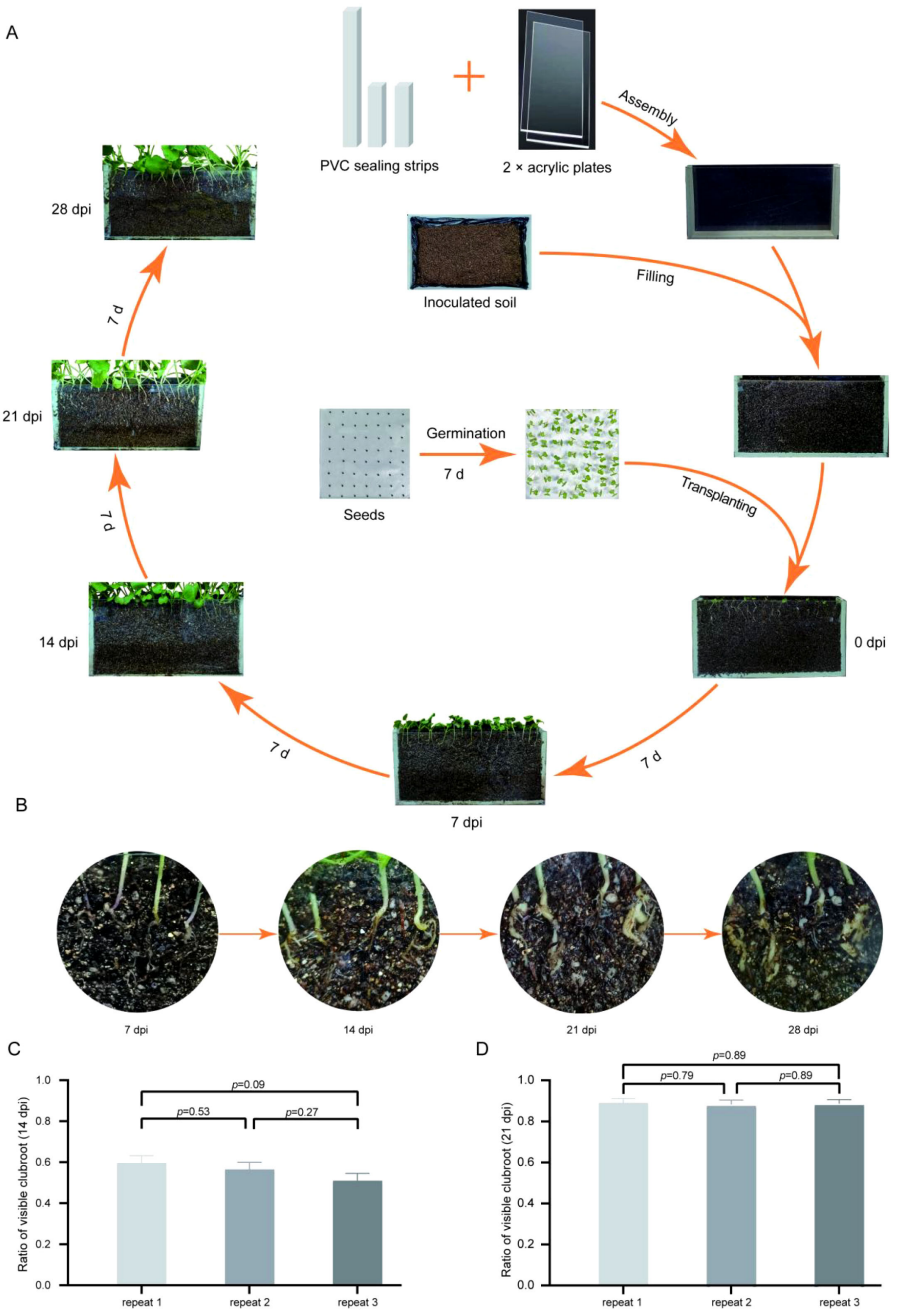
Establishment and validation of the visualization method. **(A)** Schematic diagram illustrating the establishment of the visualization method and the workflow for real-time dynamic observation. **(B)** Representative images of oilseed rape root growth at 7, 14, 21, and 28 days post-inoculation (dpi). **(C, D)** Statistical analysis of the visualized clubroot disease ratio across three biological replicates at 14 and 21 dpi. Data are shown as mean ± SD.

To identify pathogenic genes in susceptible materials and develop new disease-resistant crop germplasm, we constructed a transparent visual observation device (**Figure 1A**). The specific assembly method was as follows: two acrylic transparent plates (30 cm × 15 cm, length × width) were used as the main body, and assembled with two wide-side PVC sealing strips (14 cm × 2 cm × 1 cm, length × width × thickness) and one long-side PVC sealing strip (30 cm × 2 cm × 1 cm, length × width × thickness) for splicing and fixation.

After filling the device with *Pb*XM-infested soil, 7-day-old seedlings were planted on one side of the device, allowing real-time observation of root growth through the transparent acrylic plate. The dynamic changes in root growth of different rapeseed cultivars inoculated with *Pb*XM-infested soil were observed through acrylic transparent plates at 7, 14, 21, and 28 dpi (**Figure 1A**). Observation of the root systems at different time points post-inoculation revealed no phenotypic differences among the materials at 7 dpi. At 14 dpi, compared to CK1, the roots of CK2 exhibited macroscopically visible swelling without significant morphological distortion. By 21 dpi, root swelling in CK2 was readily apparent without the need for detailed comparison; the roots appeared “shorter and thicker” but remained within the normal root system architecture, whereas the roots of CK1 continued to exhibit normal, slender growth. At 28 dpi, the swollen morphology of CK1 roots became prominent, showing “tuberization,” which differed significantly from normal roots (**Figure 1B**). The experimental results indicate that this study, using a visualization method, observed clearly distinguishable swelling in rapeseed roots at 14 dpi with *P. brassicae*, which is the earliest time point currently reported for definitive identification of clubroot disease onset.

Since the visualization method identified disease onset—characterized by clearly distinguishable root swelling—primarily at 14 and 21 dpi, the number of plants with clubroot was recorded for 85 conventional cultivars, with three biological replicates at each time point (**Supplementary Table 2**). We performed visual analysis and statistical analysis of clubroot incidence for the 85 accessions at two time points (**Figures 1C, D**). Data analysis revealed no statistically significant differences among the three biological replicates at 14 and 21 dpi. These results demonstrated that the identification method using the transparent plate cultivation system not only enables non-destructive dynamic visualization of clubroot development but also exhibits high stability and reliability.

### 3.2 Genotypic variation of root gall developing speed

**Figure 2:**
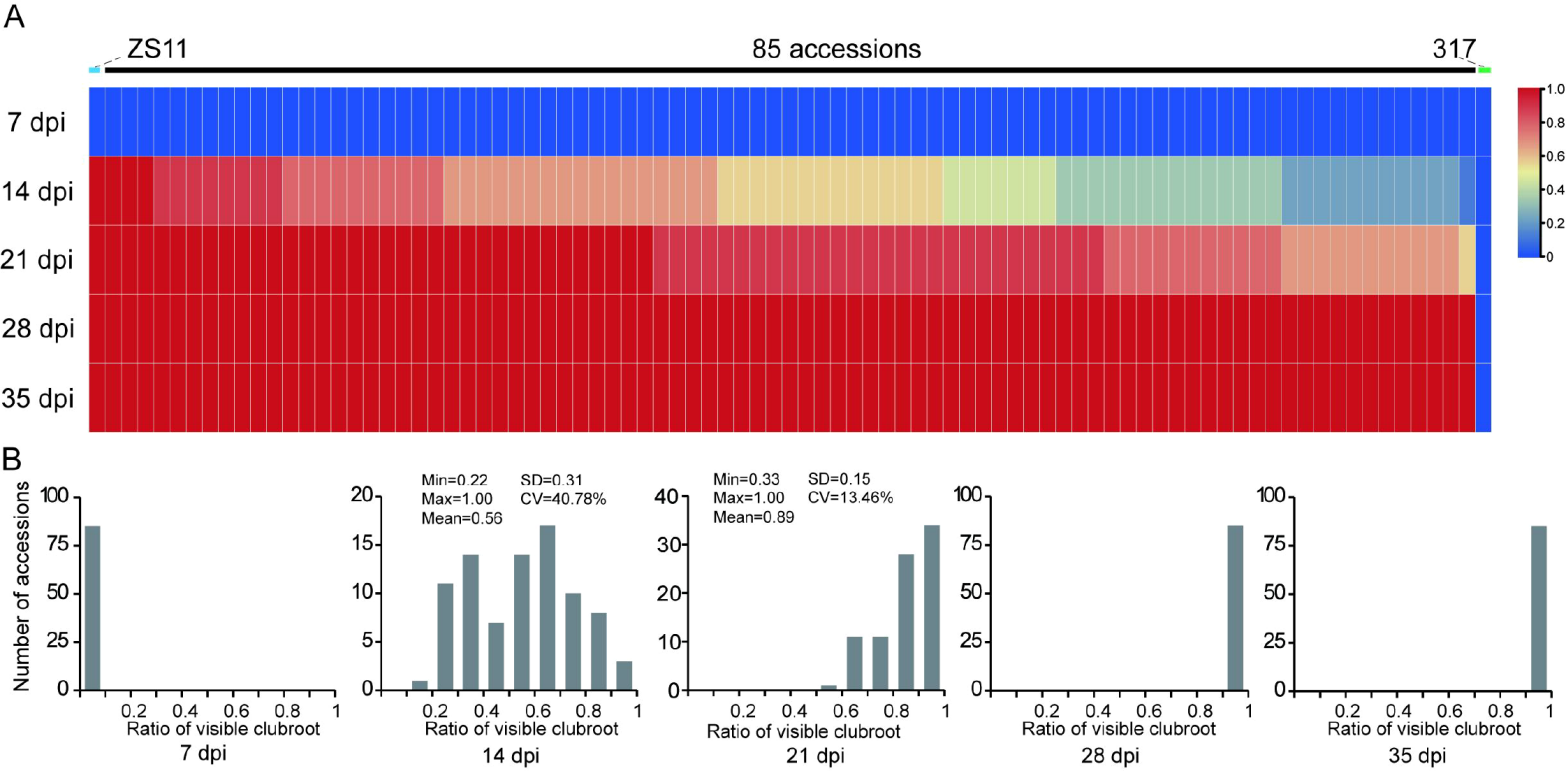
Analysis of the ratio of visible clubroot (RVC) severity in 85 germplasm resources. **(A)** Heatmap of RVC levels for each accession at 7, 14, 21, 28, and 35 days post-inoculation (dpi). ZS11 and 317 are shown as susceptible and resistant controls, respectively. **(B)** Frequency distribution of RVC across the same time points. Error bars represent standard deviation (SD); values of the coefficient of variation (CV%) are also indicated. Data are from three biological replicates (n=3 per time point).

To investigate differences in disease progression speed and severity among susceptible rapeseed germplasm, a transparent visualization method was employed. The number of visible clubroot galls on 85 core germplasm accessions was counted, the ratio of visible clubroot was calculated, and the results were plotted (**Figure 2A**; **Supplementary Table 2**). Over time, after seedling transplantation, no phenotypic differences in root morphology were observed among the 85 accessions at 7 dpi. By 14 dpi, distinguishable swelling symptoms had appeared on the roots, but significant differences in the visible clubroot ratio were noted among the 85 accessions. At 21 dpi, most varieties were fully symptomatic, exhibiting a “short and thick” root morphology while still maintaining the basic root structure. The differences in the ratio of visible clubroot among the 85 accessions had diminished by this time point. At 28 and 35 dpi, all 85 germplasm accessions were diseased, with some materials showing prominent root gall enlargement (**Figures 2A**, **1A**).

Using the RVC data from 85 germplasm accessions at 7, 14, 21, and 28 dpi, we plotted the frequency distributions of these ratios for each time point in **Figure 2B**. The results revealed that, except for ratios of 0 at 7 dpi and 1 at both 28 and 35 dpi, the rate of visible clubroot at 14 dpi ranged from 0.22 to 1.00, with a coefficient of variation (CV) of 40.78%. At 21 dpi, the disease incidence ranged from 0.33 to 1.00, with a CV of 13.46%. These findings indicate the presence of genotypic differences among the 85 germplasm accessions during infection by *P. brassicae*. The highest phenotypic variability and significant genetic diversity were observed at 14 dpi. As shown in **Figure 2B**, the distribution at 14 dpi approximated a normal distribution, suggesting that disease incidence is controlled by multiple genes, exhibits quantitative trait characteristics, and is suitable for GWAS analysis.

### 3.3 Genome-wide association study of root gall developing speed

**Figure 3:**
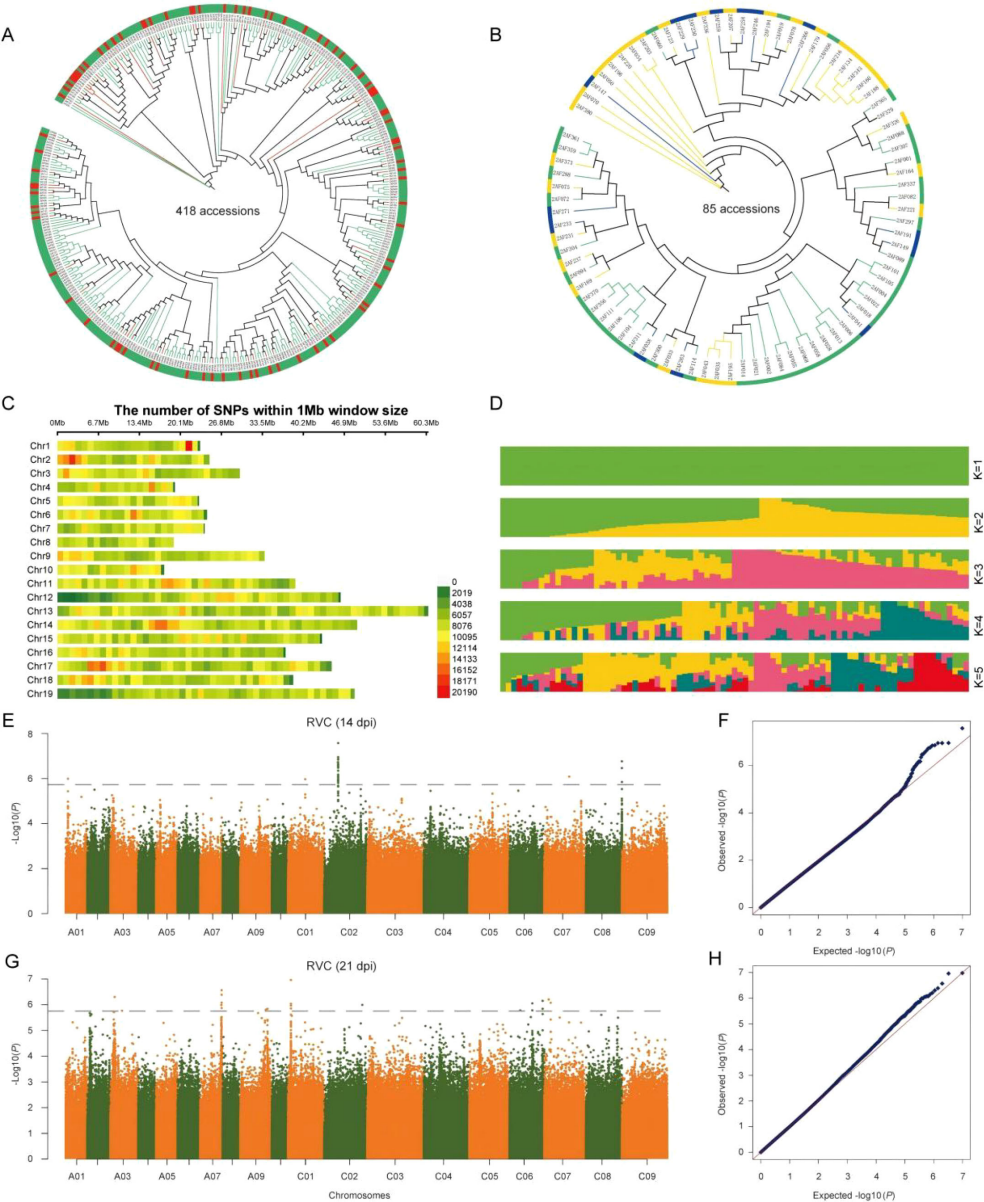
Population structure and GWAS of 85 rapeseed accessions. **(A)** Phylogenetic tree of 418 *B.napus* accessions. The outer red ring indicates the 85 core accessions used in this study, and green represents the remaining accessions. **(B)** Phylogenetic tree of the 85 rapeseed accessions. Yellow, green, and blue indicate winter-type, semi-winter-type, and spring-type accessions, respectively. **(C)** Genome-wide distribution density of SNPs across chromosomes. **(D)** Population structure analysis of 85 *B.napus* accessions. Each color represents one ancestral population; each line is represented by a vertical bar, and the length of each colored segment reflects the proportion of ancestry. The number of ancestral populations (K) was set from 1 to 5. **(E-H)** Manhattan plots and QQ-plots for ratio of visual clubroot (RVC) at 14 and 21 dpi. **(E, F)** RVC at 14 dpi; **(G, H)** RVC at 21 dpi.

A phylogenetic tree constructed from 418 publicly available accessions and the 85 accessions in this study (**Figures 3A, B**) showed that the latter represented a core germplasm collection with abundant genetic diversity and extensive geographic representation. A total of 5,287,597 SNP loci were identified across the genomes of these 85 core accessions, with an average density of one SNP per 0.12 kb (**Figure 3C**; **Supplementary Table 3**). Population structure analysis (K = 3) classified the 85 *B.napus* accessions into three subpopulations (**Figure 3D**), consisting of 31 winter-type, 39 semi-winter-type, and 15 spring-type accessions, respectively (**Supplementary Table 1**). The winter and semi-winter subpopulations exhibited numerous internal branches and scattered genetic distances, without forming tight single clusters, indicating higher genetic diversity within these two subpopulations. LD decay analysis showed that the LD decay distance was approximately 113.3 kb across all accessions, 201.2 kb for semi-winter accessions, and 327.8 kb for winter accessions, suggesting that winter-type accessions possessed higher recombination rates and greater genetic diversity. Collectively, phylogenetic relationships, population structure, and LD decay analysis confirmed that the 85 tested accessions harbored rich genetic backgrounds and high diversity levels.

To dissect the genetic factors governing the speed and severity of clubroot disease in 85 rapeseed accessions, a GWAS was performed using the mixed linear model (MLM, P + K) previously used in a study of 418 NG accessions. The RVC score at 14 and 21 dpi was used for association analysis (**Supplementary Table 4**). Manhattan plots (**Figures 3E, F**) displayed SNP loci significantly associated with RVC in rapeseed. QQ−plot analysis indicated strong natural selection effects underlying the observed phenotypic-genotypic correlations. Based on Manhattan and QQ-plot evaluations, SNPs with a significance threshold of -log_10_(P) > 5.75 were defined as significant loci. At 14 dpi, three consecutive significant peaks were detected, mainly distributed on ChrA01, ChrC02, and ChrC08, showing strong linkage disequilibrium. The phenotypic variance explained (PVE) by individual loci ranged from 1.82% to 2.37% (**Table 1**), consistent with the quantitative genetic characteristics of early root gall formation, indicating that this trait is collectively regulated by multiple minor-effect genes rather than a small number of major-effect genes. In total, 25 significant SNPs were identified, harboring 137 annotated genes. At 21 dpi, two consecutive significant peaks were observed on ChrA07 and ChrC01, also exhibiting strong LD. The PVE values of the representative loci were 2.13% and 1.85%, respectively. A total of 10 significant SNPs were detected, harboring 197 genes (**Figure 3**).

**Table 1:** Five associated loci and QTLs for RVC were detected at 14 and 21 dpi.

Trait	QTLs	Chr	Physical interval (bp)	Peak SNP	P-value	PVE (%)
14dpi	qA01	chrA01	2266140-2566140	A01_2416140	1.02E-06	1.82
14dpi	qC02	chrC02	14514075-14998476	C02_14848438	2.65E-08	2.37
14dpi	qC08	chrC08	38220584-38614415	C08_38391347	1.71E-07	2.02
21dpi	qA07	chrA07	22417993-22991575	A07_22841518	2.70E-07	2.13
21dpi	qC01	chrC01	2933564-3278753	C01_3128753	1.07E-06	1.85

These results indicate that clubroot development during root gall enlargement in *B.napus* is controlled by multiple genes with distinct temporal contributions.

Based on gene annotation and literature review, a total of 19 candidate genes associated with the early stage of clubroot disease were identified and summarized in **Table 2**. These candidate genes are widely involved in key physiological processes in *Arabidopsis*, including light response, nutrient transport, signal transduction, immune defense, and growth and development regulation. Their functions cover multiple core metabolic pathways, and some gene family members exhibit functional redundancy or synergy, jointly maintaining normal plant physiological homeostasis.

**Table 2:** Candidate genes associated with clubroot development at 14 and 21 dpi.

Traits	QTLs	Peak SNP	PVE (%)	Gene ID	Ara ID	Annotation
14dpi	qA01	A01_2416140	1.82	BnaA01g05040D	AT4G31820.1	Phototropic-responsive NPH3 family protein
				BnaA01g05280D	AT4G39030.1	MATE efflux family protein
				BnaA01g05490D	AT4G39400.1	Leucine-rich receptor-like protein kinase family protein
14dpi	qC02	C02_14848438	2.37	BnaC02g18520D	AT4G35987.1	S-adenosyl-L-methionine-dependent methyltransferases superfamily protein
				BnaC02g18590D	AT1G68810.1	basic helix-loop-helix (bHLH) DNA-binding superfamily protein
				BnaC02g18610D	AT1G68820.1	Transmembrane Fragile-X-F-associated protein
				BnaC02g18620D	NA	NA
				BnaC02g18630D	AT1G68820.1	Transmembrane Fragile-X-F-associated protein
				BnaC02g18660D	AT1G68850.1	Peroxidase superfamily protein
				BnaC02g18730D	AT1G68880.1	basic leucine-zipper 8
14dpi	qC08	C08_38391347	2.02	BnaC08g45870D	AT1G03010.1	Phototropic-responsive NPH3 family protein
				BnaC08g46010D	AT1G02740.1	MRG family protein
				BnaC08g46100D	AT1G01070.1	nodulin MtN21/EamA-like transporter family protein
21dpi	qA07	A07_22841518	2.13	BnaA07g32840D	AT1G76520.2	Auxin efflux carrier family protein
				BnaA07g32960D	AT1G76640.1	Calcium-binding EF-hand family protein
				BnaA07g32970D	AT1G76650.3	calmodulin-like 38
21dpi	qC01	C01_3128753	1.85	BnaC01g05810D	AT4G32650.1	potassium channel in Arabidopsis thaliana 3
				BnaC01g05900D	AT4G32570.1	TIFY domain protein 8
				BnaC01g05950D	AT4G32540.1	Flavin-binding monooxygenase family

According to homologous gene annotation in *Arabidopsis*, *BnaA01g05040D* and *BnaC08g45870D* both belong to the NPH3 family, which is involved in phototropism and plays critical roles in phototropin-mediated signaling pathways, participating in plant responses and adaptive regulation to blue light stimuli. Among transport-related genes, *BnaA07g32840D* encodes a member of the auxin efflux carrier family, whose core function is to mediate polar auxin transport. *BnaC01g05810D* is homologous to the potassium channel protein in *Arabidopsis*, participating in transmembrane transport of intracellular potassium ions and maintaining ion balance and cellular osmotic pressure. *BnaA01g05490D* belongs to the leucine-rich repeat receptor-like protein kinase (LRR-RLK) family. *BnaA07g32960D* and *BnaA07g32970D* are annotated as calcium-binding EF-hand family protein and calmodulin-like protein 38, respectively, with the EF-hand family being closely associated with signal transduction. Among defense- and metabolism-related genes, *BnaC02g18660D* is a member of the peroxidase superfamily and directly participates in plant disease resistance and immune responses. Among the remaining genes, members of the bHLH and bZIP8 transcription factor families are involved in transcriptional regulation, whereas proteins of the MATE efflux carrier, methyltransferase, and other families participate in fundamental metabolic processes, such as metabolite transport and methylation.

### 3.4 Comparative transcriptomic analysis of 2AF195 and 2AF058

**Figure 4:**
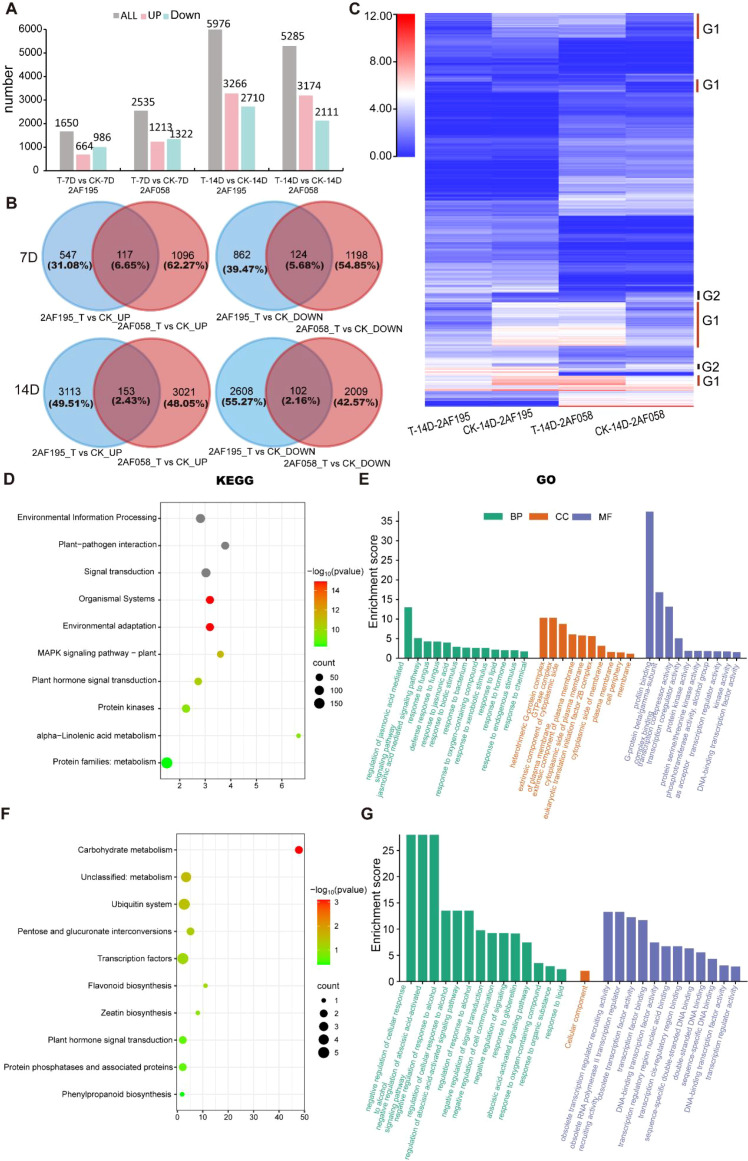
Transcriptomic analysis of 2AF195 and 2AF058 at different time points after inoculation. **(A)** Statistics of DEGs in 2AF195 and 2AF058 at 7 and 14 dpi. **(B)** Venn diagram analysis of upregulated and downregulated DEGs in the two accessions (2AF195 and 2AF058) at 7 and 14 dpi, respectively. **(C)** Heatmap of DEGs in 2AF195 and 2AF058 at 14 dpi compared with the control (CK), which were categorized into two groups (14D_G1 and 14D_G2). **(D-G)** KEGG and GO enrichment analyses of 14D_G1; F-G: those of 14D_G2. “7D” and “14D” represent samples collected at 7 and 14 dpi, respectively, while “CK” and “T” denote the application of water (control) and *Pb*XM (treatment), respectively.

To dissect the regulatory patterns of differential gene expression during the primary infection stage (0–7 dpi) and the secondary infection stage (7–24 dpi) between a slow gall-developing material 2AF195 and a fast gall-developing material 2AF058 at 14 dpi, we performed transcriptome analysis on samples from treated (T) and control (CK) groups of both materials at 7 and 14 dpi. The data showed that the number of DEGs at 14 dpi was higher than at 7 dpi for both materials (**Figure 4A**). Specifically, 2AF195 exhibited 1,650 DEGs in the T-7D vs CK-7D comparison, which increased to 5,976 DEGs in the T-14D vs CK-14D comparison. For 2AF058, the corresponding numbers were 2,535 and 5,285 DEGs, respectively (**Figure 4A**).

Venn analysis of up- and down-regulated genes from the 7- and 14-day comparisons (2AF195_T vs CK and 2AF058_T vs CK) revealed a limited number of co-expressed genes, accounting for only 2.16% to 6.65% of the total (**Figure 4B**). Expression clustering analysis of genes at 7 and 14 dpi identified opposite expression patterns at 14 dpi. Pattern 14D_G1, characterized by down-regulation in 2AF195 and up-regulation in 2AF058, comprised 1,383 genes. Pattern 14D_G2, showing the opposite trend (up-regulation in 2AF195 and down-regulation in 2AF058), contained 79 genes (**Figure 4C**; **Supplementary Tables 5**, **6**). In contrast, gene expression patterns were similar between the two materials at 7 dpi (**Supplementary Figure 1**). KEGG and GO enrichment analyses were performed on genes from 14D_G1 and 14D_G2. KEGG results indicated that 14D_G1 genes were primarily enriched in pathways related to Environmental Information Processing, Plant-pathogen interaction, Signal transduction, and the MAPK signaling pathway in plants, while 14D_G2 genes were mainly associated with Carbohydrate metabolism, the Ubiquitin system, and Transcription factors (**Figures 4D, F**). GO enrichment across the three categories (Biological Process, Cellular Component, Molecular Function) showed that the most significantly enriched terms for 14D_G1 were “regulation of jasmonic acid (JA) mediated signaling pathway,” “heterotrimeric G-protein complex,” and “heterotrimeric G-protein complex,” respectively. For 14D_G2, the top enriched terms were “negative regulation of cellular response to alcohol,” “Cellular component,” and “obsolete transcription regulator recruiting activity” (**Figures 4E, G**). These results highlight distinct functional profiles between the two clusters: 14D_G1 genes are predominantly involved in environmental adaptation, pathogen interaction, and JA regulation, whereas 14D_G2 genes are more associated with carbohydrate metabolism, ubiquitination, and cellular structure.

Data showed that the number of genes exhibiting the 14D_G1 expression pattern was significantly higher than that of genes assigned to the 14D_G2 module. Therefore, we selected the top 10% of genes with extreme differential expression values (136 genes in total, 47 of which were shared between 2AF195 and 2AF058) from the T-14D vs. CK-14D comparison of 14D_G1, along with all 79 genes in 14D_G2, for combined annotation and KEGG pathway enrichment analysis. The 14D_G1 module was mainly enriched in plant hormone signaling, disease resistance-related pathways, and pathogenicity factor-associated processes. Both shared and unique genes within this module were involved in clubroot resistance-related JA signaling pathways, including core repressors JAZ8/10 and JA/ABA-responsive transcriptional repressor WRKY40 (e.g., *BnaA06g03560D* and *BnaA03g04250D*). Additionally, this module contained disease resistance-related genes encoding nucleotide-binding site-leucine rich repeat (NBS-LRR) proteins, receptor-like kinases (RLKs), EF-hand domain-containing calcium ion receptors, and downstream calmodulin-binding proteins (e.g., *BnaA09g07720D* and *BnaC06g35270D*), as well as susceptibility factor MLO genes (e.g., *BnaAnng39980D* and *BnaCnng04240D*) that facilitate pathogen penetration of plant epidermal cells, establishment of parasitic relationships, and subsequent development of susceptible phenotypes (**Supplementary Table 7**). Genes in the 14D_G2 module were primarily associated with cell wall biogenesis and damage repair processes, including those encoding pectin methylesterases, pectate lyase superfamily proteins, and lignin biosynthesis-related enzymes involved in cell wall reinforcement and remodeling (e.g., *BnaA01g14130D*, *BnaA05g19130D*, and *BnaC03g55190D*), as well as direct defense-related wound-responsive proteins (e.g., *BnaA03g49130D* and *BnaC07g50660D*) (**Supplementary Table 8**).

Genes within these pathways exhibited significant expression divergence, with some harboring SNP or Indel variations (**Supplementary Figure 2**; **Supplementary Tables 9**, **10**). This discovery uncovers the existence of divergent regulatory networks among rapeseed materials with distinct susceptible genotypes, which provides a theoretical basis for developing novel disease-resistant germplasm by manipulating key susceptibility genes governing clubroot formation via gene-editing technologies. Furthermore, the candidate genes identified in this study represent the most promising targets for achieving this objective.

### 3.5 Candidate gene screening by combined haplotype and transcriptome analysis

**Figure 5:**
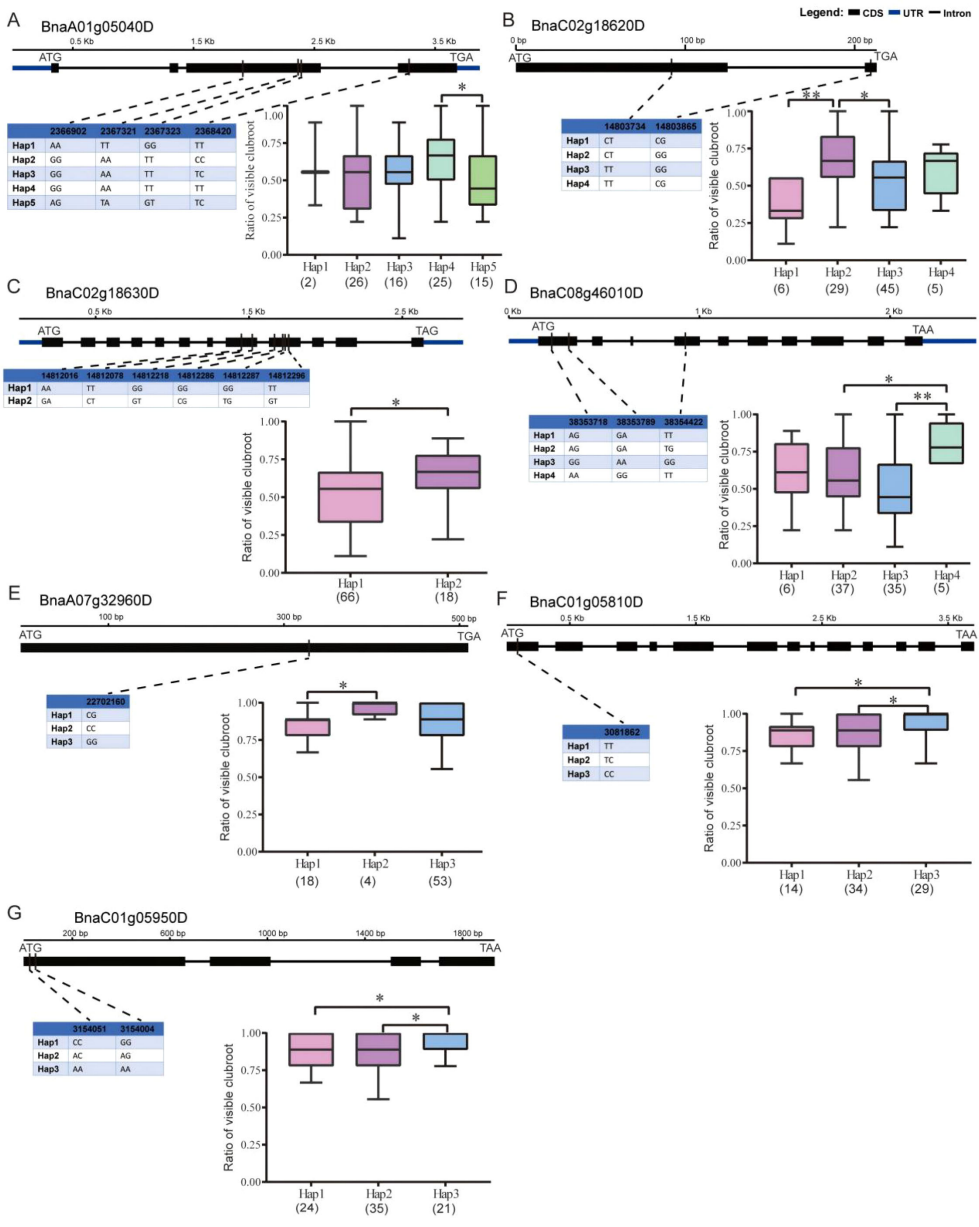
Haplotype analysis of candidate genes. [**(A–G)** Gene structure and nonsynonymous mutation site analysis of three [**(A–D)**, 14 dpi) and four [**(E–G)**, 21 dpi) candidate genes. Box plots show the visualized clubroot disease incidence at 14 or 21 dpi, categorized by haplotypes of these genes. The number of germplasms in each group is indicated below, with germplasms containing NA values excluded. Center lines, box boundaries, and whiskers represent medians, quartiles, and 1.5× interquartile ranges, respectively. *P < 0.05, **P < 0.01; two-tailed t-test.

A total of 19 candidate genes associated with early clubroot development were identified through previous GWAS. Given that nonsynonymous mutations may alter the structure or function of encoded proteins, and that changes in allele frequency may reflect selection on agronomic traits during breeding, our haplotype analysis was primarily based on nonsynonymous mutation sites in genes. Haplotype analysis revealed that nonsynonymous mutations in 7 candidate genes were significantly associated with clubroot disease phenotypes in *B.napus*. At 14 dpi, haplotypes formed by nonsynonymous mutations in *BnaA01g05040D*, *BnaC02g18620D*, *BnaC02g 18630D*, and *BnaC08g46010D* exhibited significant differences. Except for *BnaA01g05040D*, the dominant haplotypes of the other genes were all high-frequency haplotypes (e.g., Hap3 of *BnaC02g18620D*; Hap2 and Hap3 of *BnaC08g46010D*), which corresponded to low visual clubroot incidence ratios (**Figures 5A–C**). At 21 dpi, haplotypes of *BnaA07g32960D*, *BnaC01g05810D*, and *BnaC01g05950D* differed significantly. Similarly, high-frequency haplotypes (e.g., Hap1 of *BnaA07g32960D*; Hap1 and Hap2 of *BnaC01g05950D*) were associated with low clubroot incidence ratios (**Figures 5D–G**).

**Figure 6:**
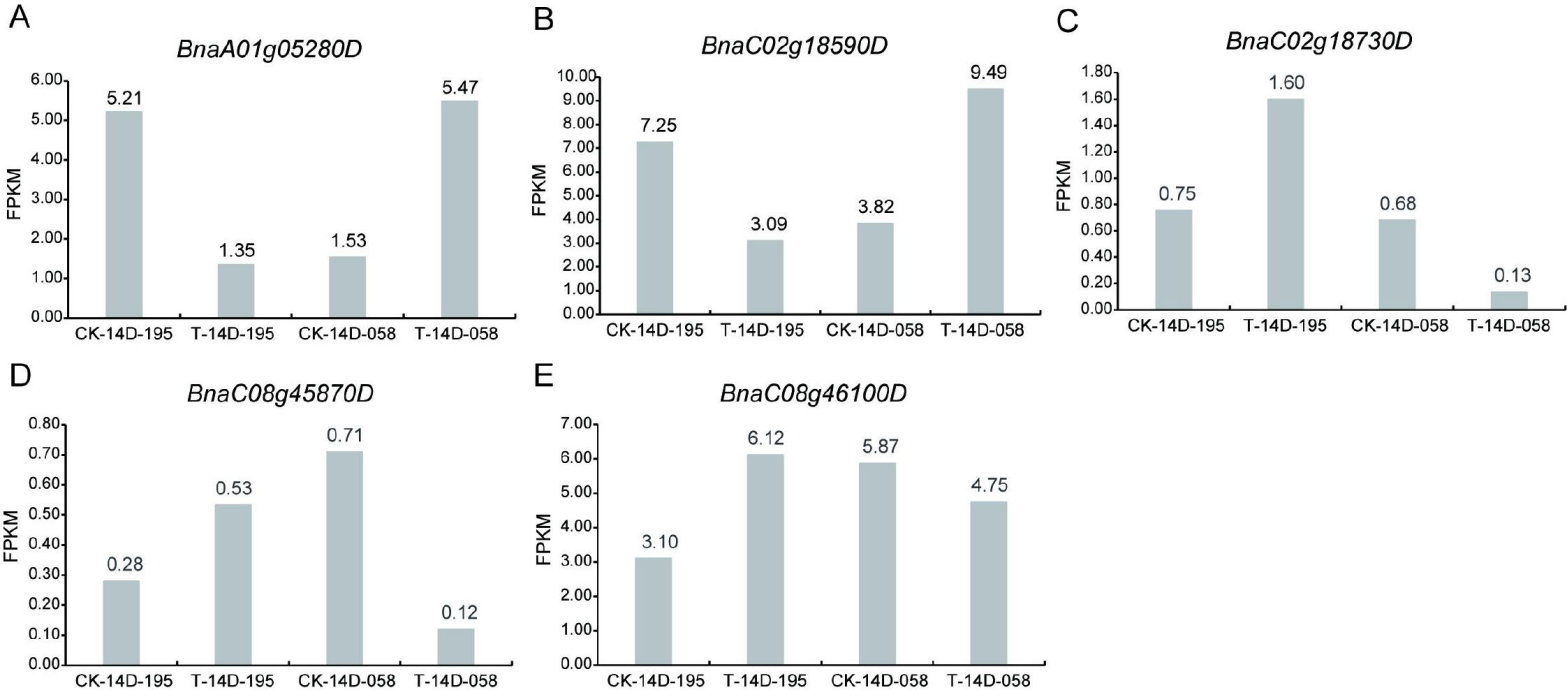
Screening and identification of significantly differentially expressed candidate genes. **(A–E)** Bar charts of FPKM expression levels for candidate genes. **(A)** *BnaA01g05040D*; **(B)** *BnaC02g18520D*; **(C)** *BnaC02g1890D*; **(D)** *BnaC02g18730D*; **(E)** *BnaC08g45870D*. The x-axis shows the 2AF195 and 2AF058 treatments and their corresponding control groups; the y-axis shows FPKM values, indicating the transcriptional expression levels of each gene.

Among them, 13 candidate genes were identified at 14 dpi. Combined with transcriptomic expression data, five differentially expressed genes between delayed-infection and early-infection lines at 14 dpi were further screened and identified as core key candidate genes. As shown in **Figure 6**, the expression levels of *BnaA01g05280D* and *BnaC02g18590D* were downregulated under 2AF195 treatment but upregulated under 2AF058 treatment. By contrast, *BnaC02g18730D*, *BnaC08g45870D*, and *BnaC08g46100D* were up regulated in 2AF195 but down regulated in 2AF058. Notably, *BnaC08g46100D*, a MtN21 family nodulin gene, was also identified by haplotype analysis of candidate genes, indicating that this gene is closely associated with clubroot resistance at both the population and expression levels, thereby supporting its greater reliability.

## 4 Discussion

### 4.1 Advantages of the transparent plate visualization method

In current research on disease resistance in crops such as rapeseed, *B.rapa*, and *B.oleracea*, studies on clubroot, caused by *Pb*XM, are indispensable. Whether screening for disease-resistant germplasm, identifying physiological races, or breeding resistant varieties, all depend on phenotypic assessment of clubroot to attain research objectives. The central role of this process has been validated by Zamani-Noor and Jędryczka, who, in their systematic review of global systems for differentiating *P.brassicae* races (e.g., the Williams system and Canadian differential host sets), explicitly noted that phenotypic evaluation serves as the critical link connecting pathogen pathogenicity and host resistance (Zamani-Noor and Jędryczka, 2024).

Traditionally, phenotypic assessments in both domestic and international studies have relied on conventional cultivation practices, such as field or pot planting; however, these approaches exhibit significant limitations in clubroot identification. First, they lack the capacity for intuitive dynamic visualization. Observing root disease progression necessitates destructive sampling (e.g., uprooting and washing), which aligns with Stefanowicz et al.’s research on *P. brassicae*-induced root cell enlargement. Their work demonstrated that clubroot-associated root morphological changes require direct observation, a requirement frustrated by the destructive nature of traditional methods—these methods prevent continuous monitoring of clubroot development in the same specimens across multiple time points (Stefanowicz et al., 2021). Second, the phenotypic assessment period is prolonged, usually exceeding 6 weeks. This aligns with the temporal characteristics of the traditional differential host method observed by Zamani-Noor and Jędryczka in multiple studies, which remains the standard for confirming clubroot phenotypes (Zamani-Noor and Jędryczka, 2024).

To address these challenges, there is an urgent need to develop novel methods for clubroot evaluation. Accordingly, we have established a rapid, efficient, visually accessible, and dynamically monitorable clubroot assessment system. This method employs transparent plate cultivation to achieve non-destructive, time- and labor-efficient evaluation, accommodating 15 plants per plate while enabling high-throughput analysis, real-time dynamic visualization, and convenient leaf sampling. Its non-invasive detection principle aligns with the automated phenotyping logic of the BluVision Micro imaging platform developed by Lück et al., both of which aim to capture phenotypic details without disrupting plant growth (Lück et al., 2025). Moreover, this system ensures reliable individual-plant resistance/susceptibility assessment and exhibits high consistency in disease incidence across replicated experiments, providing a fully visualizable technical platform for clubroot research. This breakthrough has significant implications for phenotypic screening and disease-resistant breeding: Fredua-Agyeman et al. emphasized, in their investigation of resistance breakdown in the European oilseed rape cultivar ‘Tosca,’ that dynamic matching between resistance genes and physiological races relies on efficient phenotypic validation (Fredua-Agyeman et al., 2021). Thus, our method provides critical technical support for accelerating the discovery of resistant germplasm and the development of cultivars.

### 4.2 GWAS population analysis and mechanistic prediction of key candidate gene

Although this study identified valuable QTLs controlling early root gall formation, several limitations should be noted. The relatively small GWAS population may weaken the detection of minor-effect genetic loci and limit mapping resolution, a common constraint in association analyses of crop disease resistance (Zatybekov et al., 2020). Further studies with larger germplasm panels are required to validate these loci and improve genetic mapping accuracy. Compared with previous rapeseed clubroot GWAS, most earlier studies concentrated on late-stage disease resistance and major resistance loci (Li et al., 2016). In contrast, the present work focuses on the early stage of gall initiation, and the identified QTLs are distinct from previously reported intervals. These findings provide novel genetic clues for understanding the early interaction between Brassica napus and Plasmodiophora brassicae.

Notably, BnaC08g46100D was prioritized as the core candidate gene within the target interval. This gene belongs to the MtN21 transporter family, which confers obvious functional advantages over other co-localized genes. Most adjacent genes lack definite annotation for biotic stress response, whereas MtN21 family members are well documented to mediate transmembrane transport of nutrients and signaling molecules during plant biotic stress (Ma et al., 2006; Garcia et al., 2023).

These results suggest that MtENODL29 participates in membrane lipid modification and transport by interacting with MtnsLTP, MtKCR, and MtSec61γ, facilitating the formation of symbiosome membranes as the alfalfa rhizobium (Sinorhizobium meliloti) strain 1021 is released into nodule cells (Wang et al., 2026). During Plasmodiophora brassicae infection, BnaC08g46100D may regulate membrane lipid modification and lipid transport, thereby facilitating the intracellular colonization and proliferation of clubroot pathogens. In summary, the conserved transport capacity of the MtN21 family supports this gene as the most promising candidate for subsequent functional validation. CRISPR-mediated knockout and allele swapping will be performed to verify their effects on the incidence and severity of clubroot gall formation.

### 4.3 Correlations of JA pathway, MLO-related susceptibility, and cell wall remodeling with gall development

This study compared the differentially expressed genes at key infection stages of *P.brassicae* between the delayed-disease line 2AF195 and the susceptible line 2AF058. Functional enrichment analysis focused on plant hormone signaling, disease susceptibility responses, cell wall remodeling, and damage repair. These findings provide critical insights into the regulatory mechanisms underlying clubroot resistance in *B.napus* and offer valuable genetic resources for disease-resistance breeding.

The JA signaling pathway serves as a key defense pathway in plants against infections by fungi, oomycetes, and other pathogens, and the expression regulation of JAZ family proteins—core repressors of JA signaling—directly influences the intensity of disease resistance responses (Aubert et al., 2015; Ding et al., 2022; Zhou et al., 2025). In this study, JAZ8/10 and WRKY40 (a transcriptional repressor of JA/ABA signaling) were downregulated in 2AF195 but upregulated in 2AF058. Meanwhile, cytochrome P450 genes that promote JA signal attenuation showed a similar expression trend. These findings suggest that the resistant genotype 2AF195 may maintain sustained activation of the JA pathway by downregulating JA signal repressors and reducing JA signal attenuation, thereby enhancing defense responses. In contrast, the upregulation of JA signal repressors in the susceptible genotype 2AF058 may weaken the defense pathway, facilitating successful pathogen colonization. This is consistent with the conclusion proposed by Chen et al. that “suppression of JA responses helps *T. thlaspeos* maintain an extended biotrophic phase and asymptomatic colonization” differential expression of disease resistance-related genes in the 14D_G1 module further confirms the resistance divergence between the two genotypes. Notably, the susceptibility factor *MLO* was upregulated in 2AF058. *MLO* genes have been confirmed as key regulatory factors for susceptibility in various crops, and their activation promotes pathogen penetration of plant epidermal cells and the establishment of parasitic relationships (Jrgensen, 1992; Kuhn et al., 2017; Kusch and Panstruga, 2017; Huebbers et al., 2024), which may be one of the important reasons for the obvious susceptible phenotype of 2AF058.

The plant cell wall serves as not only as an effective physical barrier against various biotic stresses but also as a key component of the plant innate immune system (Cantu et al., 2008; Molina et al., 2024). In this study, cell wall-related genes in this module—such as pectin methylesterase, pectin lyase superfamily proteins, xyloglucan endotransglycosylase, and lignin biosynthesis-related genes—exhibited differential expression between the two genotypes. The activation of lignin biosynthesis-related genes can promote lignin deposition, which is part of the basal defense response (Höch et al., 2021; Xia et al., 2024). Studies have reported that activation of the broad-spectrum resistance gene *WTS* (WeiTsing) induces the expression of multiple plant defense-related genes, particularly upregulating the levels of receptor-like proteins (RLPs) and cell wall-associated kinases (WAKs) (Wang et al., 2023). These results suggest that 2AF195 may construct an efficient physical defense system by enhancing cell wall reinforcement and pathogen perception capacity. In contrast, insufficient expression regulation of cell wall-related genes in 2AF058 may result in a weak physical barrier, rendering it unable to effectively resist pathogen invasion.

To date, numerous studies have confirmed extensive direct and indirect associations between JA signaling and cell wall metabolism. Denness et al. (Cell Wall Damage-Induced Lignin Biosynthesis Is Regulated by a Reactive Oxygen Species- and Jasmonic Acid-Dependent Process in Arabidopsis) revealed in Arabidopsis that cell wall damage regulates lignin biosynthesis through the dynamic crosstalk between JA and ROS, thereby activating the JA pathway and initiating defensive cell wall reinforcement (Denness et al., 2011). Im et al. (Jasmonate activates secondary cell wallbiosynthesis through MYC2-MYB46 module.) further demonstrated that JA activates secondary cell wall biosynthesis via the MYC2-MYB46 module, which significantly induces the transcription and protein accumulation of MYB46 and upregulates the expression of downstream cell wall synthesis-related genes (Im et al., 2024). Accumulated evidence also indicates that JA enhances cell wall mechanical strength and plant disease resistance by promoting lignin deposition and the accumulation of cell wall structural proteins. Collectively, JA signaling exhibits close crosstalk with cell wall remodeling and plays a vital regulatory role in plant defense responses.

This study integrates transparent phenotyping, GWAS, and transcriptomic analysis to partially elucidate the molecular mechanisms underlying *B.napus* response to *P.brassicae* infection. However, conventional bulk transcriptomics fails to capture root cell spatiotemporal responses and cannot fully uncover the fine regulatory mechanisms governing gall development. In contrast, single-cell and spatial transcriptomics enable cell-type-specific dissection of plant pathogen responses (Ali et al., 2026), thereby refining the regulatory network underlying gall formation. Furthermore, redox homeostasis and defense responses are critical for plant disease resistance and susceptibility, with ROS/RNS acting as core signaling molecules involved in pathogen perception and abnormal tissue development (Ali et al., 2025). Collectively, advances in redox regulation help clarify the roles of the JA pathway, cell wall remodeling, and susceptibility-related modules during gall development, providing comprehensive theoretical support for this study’s findings.

## Data Availability

The data presented in the study are deposited in the China National Center for Bioinformation (CNCB) repository, accession number PRJCA065035.

## Supplementary material

Supplementary Figure 1 Gene Expression Patterns of 2AF195 and 2AF058 in Treatment and Control Groups at 7 days post-inoculation (dpi).

Supplementary Figure 2 Gene Annotations & Expression Levels: Modules 14D_G1 and 14D_G2. A, B: Gene Annotations and Expression Levels of Genes in Modules 14D_G1 and 14D_G2, Respectively.

Supplementary Table 1 Summary of 85 accessions for genome re-sequencing in this study.

Supplementary Table 2 Disease incidence data of 85 rapeseed accessions at various time points following inoculation with the Xinmin strain.

Supplementary Table 3 SNP density distribution across chromosomes.

Supplementary Table 4 Candidate genes within QTL intervals identified by GWAS.

Supplementary Table 5 1383 Genes in Module 14D_G1 and Their Expression Levels.

Supplementary Table 6 79 Genes in Module 14D_G2 and Their Expression Levels.

Supplementary Table 7 Top 10% DEGs (T-14D vs CK-14D) in 2AF195/2AF058 (Module 14D_G1): Expression Levels & KEGG Pathways (Shared/Specific Genes).

Supplementary Table 8 79 Genes in Module 14D_G2: Expression Levels & KEGG Pathways.

Supplementary Table 9 Gene Expression Levels of Genes Within the annotation of Modules 14D_G1.

Supplementary Table 10 Gene Expression Levels of Genes within the Pathways of Modules 14D_G2.
