# Supplementary material for: Identification of candidate genes involved in root gall formation during early infection of Plasmodiophora brassicae in B.napus
Source: Front Plant Sci. 2026 Jun 10;17:1847458. doi: 10.3389/fpls.2026.1847458 (PMC13291114; doi:10.3389/fpls.2026.1847458)
Supplement: Supplementary Figure 1 — Gene Expression Patterns of 2AF195 and 2AF058 in Treatment and Control Groups at 7 days post-inoculation (dpi). [file Table1.docx]

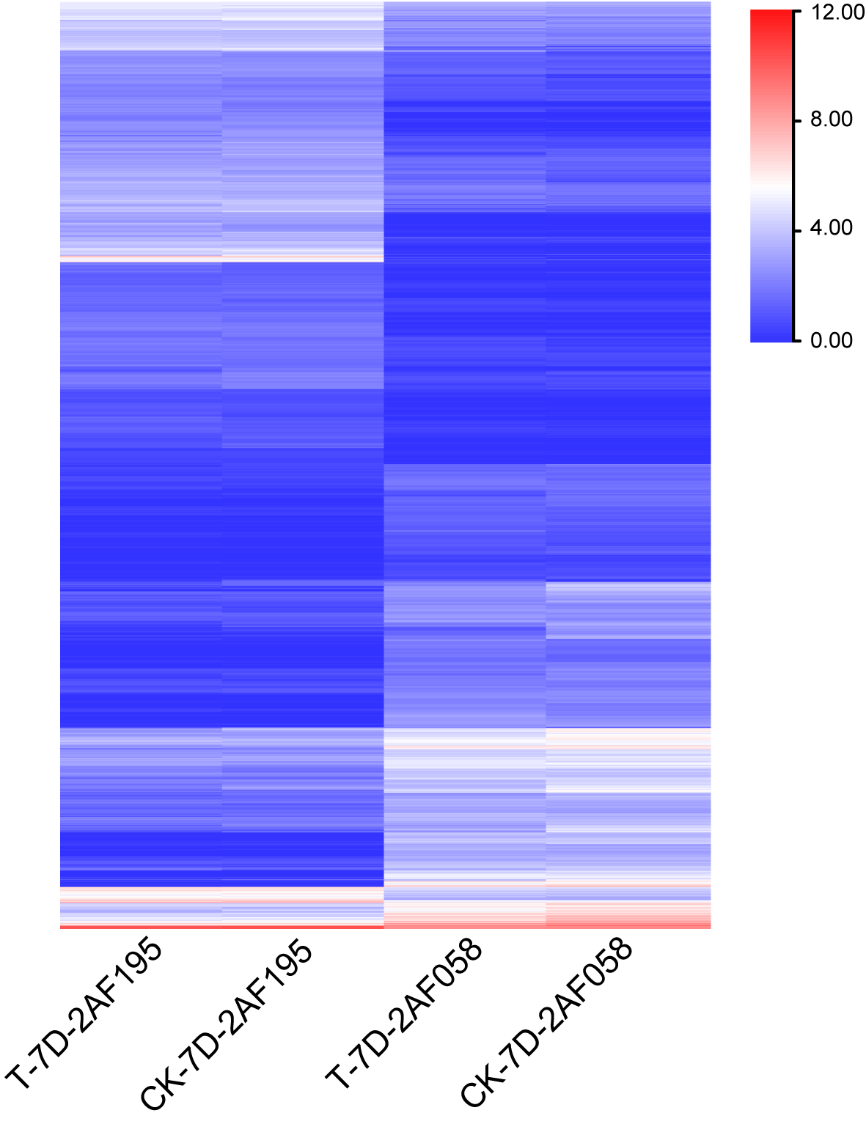


**Fig S1** Gene Expression Patterns of 2AF195 and 2AF058 in Treatment and Control Groups at 7 Days Post-Inoculation(DPI).


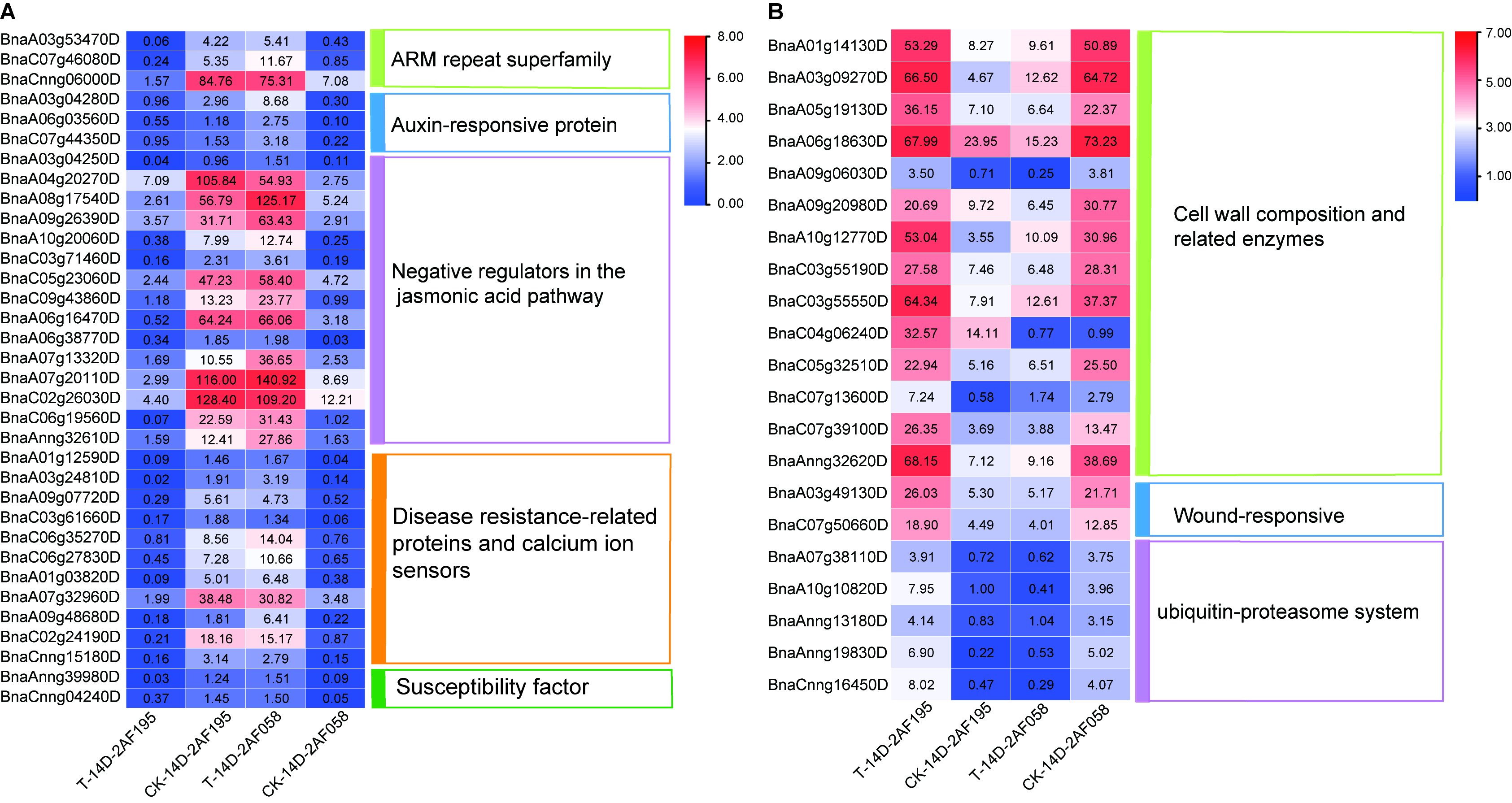


**Fig S2** Gene Annotations & Expression Levels: Modules 14D_G1 and 14D_G2. A, B: Gene Annotations and Expression Levels of Genes in Modules 14D_G1 and 14D_G2, Respectively.
